# Inhibitory Efficacy of Main Components of *Scutellaria baicalensis* on the Interaction between Spike Protein of SARS-CoV-2 and Human Angiotensin-Converting Enzyme II

**DOI:** 10.3390/ijms25052935

**Published:** 2024-03-02

**Authors:** Cheng-Han Lin, Ho-Ju Chang, Meng-Wei Lin, Xin-Rui Yang, Che-Hsiung Lee, Chih-Sheng Lin

**Affiliations:** 1 Department of Biological Science and Technology, National Yang Ming Chiao Tung University, Hsinchu 30068, Taiwan; a0975273923@gmail.com (C.-H.L.); imhrchang@gmail.com (H.-J.C.); lmw1018@nycu.edu.tw (M.-W.L.); yllas0315@gmail.com (X.-R.Y.); ikemanleee@gmail.com (C.-H.L.); 2 Department of Plastic and Reconstructive Surgery, Chang Gung Memorial Hospital, Linkou, Taoyuan 333423, Taiwan; 3 Center for Intelligent Drug Systems and Smart Bio-Devices (IDS2B), National Yang Ming Chiao Tung University, Hsinchu 30068, Taiwan

**Keywords:** SARS-CoV-2, angiotensin-converting enzyme 2 (ACE2), spike protein, traditional Chinese medicine, *Scutellaria baicalensis*, baicalein, baicalin

## Abstract

Blocking the interaction between the SARS-CoV-2 spike protein and the human angiotensin-converting enzyme II (hACE2) protein serves as a therapeutic strategy for treating COVID-19. Traditional Chinese medicine (TCM) treatments containing bioactive products could alleviate the symptoms of severe COVID-19. However, the emergence of SARS-CoV-2 variants has complicated the process of developing broad-spectrum drugs. As such, the aim of this study was to explore the efficacy of TCM treatments against SARS-CoV-2 variants through targeting the interaction of the viral spike protein with the hACE2 receptor. Antiviral activity was systematically evaluated using a pseudovirus system. *Scutellaria baicalensis* (*S. baicalensis*) was found to be effective against SARS-CoV-2 infection, as it mediated the interaction between the viral spike protein and the hACE2 protein. Moreover, the active molecules of *S. baicalensis* were identified and analyzed. Baicalein and baicalin, a flavone and a flavone glycoside found in *S. baicalensis*, respectively, exhibited strong inhibitory activities targeting the viral spike protein and the hACE2 protein, respectively. Under optimized conditions, virus infection was inhibited by 98% via baicalein-treated pseudovirus and baicalin-treated hACE2. In summary, we identified the potential SARS-CoV-2 inhibitors from *S. baicalensis* that mediate the interaction between the Omicron spike protein and the hACE2 receptor. Future studies on the therapeutic application of baicalein and baicalin against SARS-CoV-2 variants are needed.

## 1. Introduction

The coronavirus disease 2019 (COVID-19) pandemic, caused by severe acute respiratory syndrome coronavirus 2 (SARS-CoV-2), posed a variety of threats and challenges to public healthcare systems [1,2,3]. As the pandemic progressed, SARS-CoV-2 mutated into highly infective strains such as B.1.617.2 (Delta) and BA.2 (Omicron). The emergence of these variants complicated the development of broad-spectrum vaccines and drugs [4]. Therefore, it is important to search for approved compounds with broad-spectrum antiviral activity against SARS-CoV-2.

SARS-CoV-2 is a positive-sense, single-stranded, enveloped RNA virus. SARS-CoV-2 contains four major structural proteins, which include the spike (S), envelope (E), membrane (M), and nucleocapsid genes (N) [5,6,7]. Rod-shaped protrusions and trimeric spike proteins characterize the surface of the SARS-CoV-2 virus. The SARS-CoV-2 spike protein binds to the host cell receptor, human angiotensin-converting enzyme 2 (hACE2), facilitating conformational changes and virus entry [8,9,10,11]. As a result, blocking the interaction between the viral spike protein and hACE2 is a potential therapeutic strategy against COVID-19.

Traditional Chinese medicine (TCM) represents a system of treatment protocols dating back thousands of years, which promote health, healing, longevity, and complex healthcare [12,13]. TCM has also contributed to COVID-19 treatment [14,15]. Taiwan Chingguan-Yihau (NRICM101), a TCM formula targeting viral respiratory infection and immunomodulation reported by the National Research Institute of Chinese Medicine, Taiwan (NRICM), has been commonly recommended and was clinically proven to be effective for the treatment of COVID-19 [16,17,18]. Based on the theoretical TCM system, the functions of *Scutellaria baicalensis* (*S. baicalensis*), *Houttuynia cordata* (*H. cordata*), and *Isatis indigotica* (*I. indigotica*) in NRICM101 are to clear heat, decrease dampness, promote diuresis, detoxify, and reduce swelling [19,20,21]. In addition to their traditional uses, *S. baicalensis*, *H. cordata*, and *I. indigotica* have been extensively studied for their pharmacological effects against COVID-19 [22,23,24,25]; the findings of such studies provide promising avenues for the development of antiviral therapies.

This study aimed to discover which active compounds that are used in TCM disrupt the interaction between the SARS-CoV-2 spike protein and hACE2 to inhibit infection. The inhibitory activities were evaluated against SARS-CoV-2 pseudovirus infection. We investigated the inhibitory efficacy of the main components of *S. baicalensis* on the interaction between the SARS-CoV-2 spike protein and hACE2.

## 2. Results

### 2.1. Establishment of a Pseudovirus System for Evaluating the Binding Efficacy of Viral Spike Protein and hACE2 Receptor

**Figure 1:**
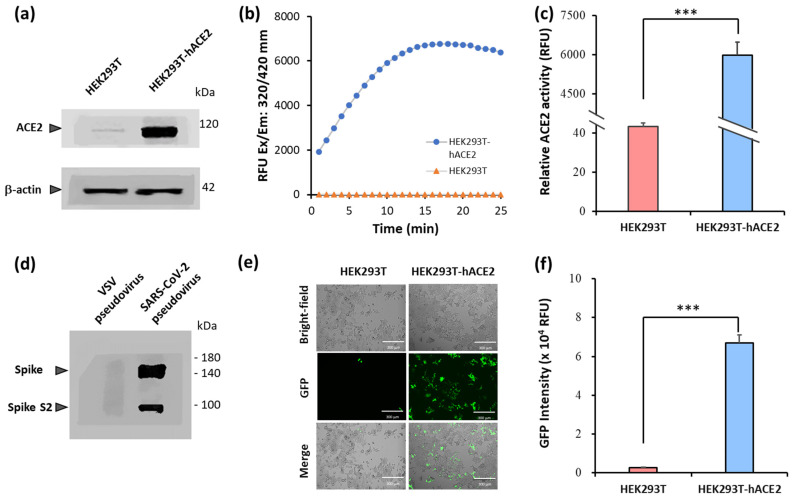
Establishment of SARS-CoV-2 spike pseudovirus system. Protein expression of hACE2 in HEK293T-hACE2 and HEK293T cells was examined via Western blotting analysis (**a**). ACE2 activity in HEK293T-hACE2 and HEK293T cells was examined using ACE2 activity assays (**b**). GFP intensity in HEK293T-hACE2 and HEK293T cells was quantified using a Synergy HT multimode microplate reader (**c**). The spike protein expression of SARS-CoV-2 spike pseudovirus and VSV pseudovirus was examined via Western blotting (**d**). GFP images represent HEK293T-hACE2 and HEK293T cells infected with SARS-CoV-2 spike pseudovirus (**e**). GFP intensity in HEK293T-hACE2 and HEK293T cells was quantified (**f**). All values are expressed as the mean ± SD (*n* = 5) for each group. The statistical comparison between groups was conducted using Student’s *t*-test; *** *p* < 0.001 vs. HEK293T cells.

A pseudovirus system was established to evaluate the binding efficacy of the viral spike protein to the hACE2 receptor. In the pseudovirus system, HEK293T cells stably expressing hACE2, named HEK293T-hACE2, were infected with a SARS-CoV-2 spike pseudovirus. The binding efficacy was quantified using the green fluorescent protein (GFP) intensity of the pseudovirus-infected cells. To characterize the hACE2 in the cells, the protein expression and enzyme activity of hACE2 were measured through Western blot and enzyme activity assays, respectively. The protein expression of ACE2 in HEK293T-hACE2 cells was significantly higher than that in HEK293T cells (Figure 1a). The results in Figure 1b,c show that the hACE2 activity level was higher (6000 RFU) in HEK293T-hACE2 cells than in HEK293T cells (44 RFU). These results indicate the increased expression of hACE2 in HEK293T-hACE2 cells compared with that in HEK293T cells (*p* < 0.001).

A SARS-CoV-2 spike pseudovirus with the spike protein on its surface was produced. To verify the incorporation of the spike protein, the surface protein of the pseudovirus was analyzed via Western blotting. The results in Figure 1d show that specific bands were found in the lanes of the SARS-CoV-2 spike pseudovirus, but no specific band was found in the VSV pseudovirus in the corresponding position. Following the pseudovirus’s production, HEK293T and HEK293T-hACE2 cells were infected with the SARS-CoV-2 spike pseudovirus to examine the efficacy with which the viral spike protein bound to cellular hACE2. A significant difference was observed between the HEK293T and HEK293T-hACE2 cells (*p* < 0.001) (Figure 1e). The GFP intensity in HEK293T-hACE2 cells was 6 × 10^4^ RFU, in comparison to the 5 × 10^3^ RFU in HEK293T cells (Figure 1f). Overall, the results demonstrated in Figure 1 confirm that hACE2 was overexpressed in HEK293T-hACE2 cells. Additionally, the established SARS-CoV-2 spike pseudovirus efficiently infected HEK293T-hACE2 cells through the interaction between the viral spike protein and the hACE2 receptor.

### 2.2. Identification of SARS-CoV-2 Spike Pseudovirus System for Drug Candidates

**Figure 2:**
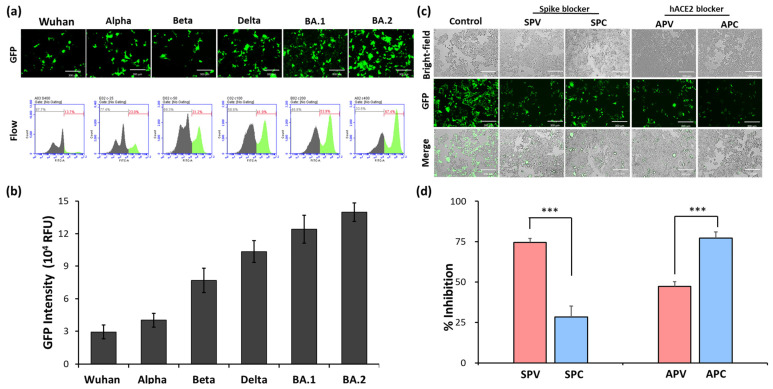
Infection efficiency of SARS-CoV-2 spike variant pseudovirus and inhibitory efficacy of spike and hACE2 blockers. GFP images and flow cytometry analysis represent HEK293T-hACE2 cells that were infected with pseudoviruses expressing the spike proteins of Wuhan, Alpha, Beta, Delta, BA.1, and BA.2. strains (**a**). GFP intensity was detected with a Synergy HT multimode microplate reader (**b**). GFP images of HEK293T-hACE2 cells infected with SARS-CoV-2 BA.2 spike pseudovirus (**c**). The inhibitory efficacy of spike and hACE2 blockers preincubated with pseudovirus or cells (**d**). All values are expressed as the mean ± SD (*n* = 5) for each group. The statistical comparison among groups was conducted using Student’s *t*-test; *** *p* < 0.001 vs. SPV or APV group. SPV, SPC, APV, and APC are defined in Table 1.

RNA recombination occurs at a high rate in coronaviruses, increasing the virus’s plasticity for mutation. SARS-CoV-2 variants were found to increase transmissibility and strengthen the ability to escape immunity. Variants of the spike-protein-expressing pseudovirus, including Wuhan, Alpha, Beta, Gamma, Delta, and Omicron BA.1, as well as BA.2, were established in this study. As shown in Figure 2, the highest GFP intensity was observed in the Omicron BA.2 variant, meaning that the Omicron BA.2 pseudovirus has a better infection efficiency than the other variants. To fight against current and future SARS-CoV-2 variants, we used the Omicron BA.2 variant for the identification of SARS-CoV-2 antiviral drug candidates.

Blocking the interaction between the viral spike protein and cellular hACE2 can be used as a therapy to treat COVID-19. The reproducibility of the pseudovirus system was examined using two typical blockers, a viral spike blocker (cysteamine) [26,27] and an hACE2 blocker (Dalbavancin) [28,29] (Table 1). HEK293T-hACE2 cells were pretreated with a putative viral spike blocker and a putative hACE2 blocker for 4 h, and then the cells were infected with the pseudovirus (SPC and APC groups, respectively). The BA.2 pseudovirus was pretreated with a spike blocker and an hACE2 blocker for 4 h and then used to infect HEK293T-hACE2 cells (SPV and APV groups, respectively). The inhibitory activities of the viral spike blocker and hACE2 blocker are shown in Figure 2c. The inhibitory values of the SPC, SPV, APC, and APV groups were 27.9, 74.2, 76.8, and 51.8%, respectively. The results (Figure 2d) showed that the spike and hACE2 blockers inhibited the pseudovirus’s entry into the cells. Collectively, these data indicate that the pseudovirus system was successfully established for the evaluation of candidate drugs and therapeutics targeting the virus or host cells.

### 2.3. Assessment of Inhibitory Efficacy of Herbs on Pseudovirus Infection

**Figure 3:**
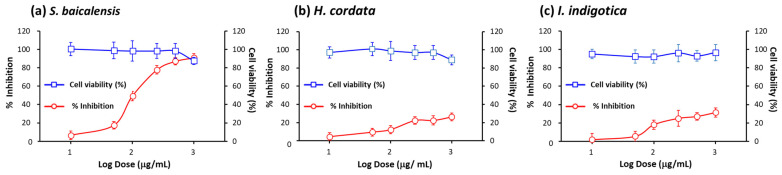
Assessment of inhibitory efficacy of *S. baicalensis*, *H*. *cordata*, and *I*. *indigotica* against SARS-CoV-2 BA.2 spike pseudovirus infection. The inhibitory efficacy and cell viability of *S. baicalensis* (**a**), *H. cordata* (**b**), and *I. indigotica* (**c**) were quantified using a Synergy HT multimode microplate reader at final concentrations of 0, 10, 100, 250, 500, and 1000 μg/mL for 24 h. All values are expressed as mean ± SD for each group (*n* = 5).

We focused on finding the biologically active components in NRICM101. The *S. baicalensis*, *H. cordata*, and *I. indigotica* species used in NRICM101 demonstrated the ability to fight against SARS-CoV-2 infection. The developed pseudovirus system was used to detect the inhibitory effects of *S. baicalensis*, *H. cordata*, and *I. indigotica* on the interaction between the viral spike protein and the hACE2 receptor. First, we analyzed the effect of different concentrations of *S. baicalensis*, *H. cordata*, and *I. indigotica* on cell viability using an MTT assay. The results showed that the cell viability slightly decreased with *S. baicalensis* and *H. cordata* treatment at 1000 μg/mL (Figure 3a,b). When the cells were treated with 1000 μg/mL of *I. indigotica*, there was a similar decrease in cell viability compared with 10 μg/mL of *I. indigotica* (Figure 3c). These substances showed low cytotoxicity within 1000 μg/mL. Collectively, the cell viability and efficacy data for the pseudovirus indicated that *S. baicalensis* (250 μg/mL) showed significantly more effective inhibitory efficacy compared to *H. cordata* and *I. indigotica* (250 μg/mL). This result indicates that *S. baicalensis* plays an important role in NRICM101, blocking the interaction between the viral spike protein and the hACE2 receptor.

### 2.4. Identification of Active Ingredients in S. baicalensis

**Figure 4:**
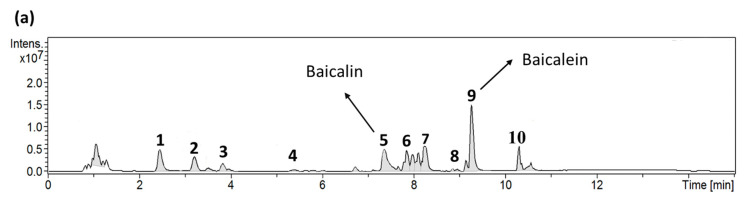

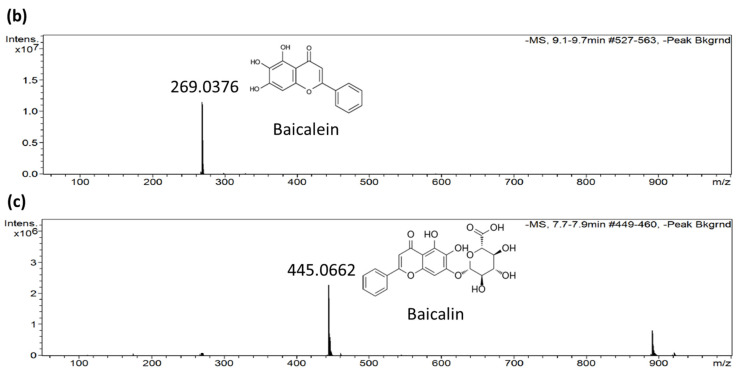
LC-MS fingerprint profiles of *S. baicalensis* extract. LC profiles of the TCM compound were obtained with an Impact HD Q-TOF mass spectrometer through ESI/MS experiments. Ten major constituents were found in the *S. baicalensis* extract (**a**). ESI/MS negative-mode mass spectra fingerprint of baicalein (**b**). ESI/MS negative-mode mass spectra fingerprint of baicalin (**c**).

*S. baicalensis* effectively inhibited the interaction between the viral spike protein and hACE2. The relative inhibition ratio was determined using the developed pseudovirus system. Then, we focused on finding the biologically active components in *S. baicalensis*. We used LC-MS to gradually separate the compounds in *S. baicalensis* and track the biologically active ingredients. The 10 major active ingredients in *S. baicalensis* were identified as follows: chrysin 6-*C*-arabinoside-8-*C*-glucoside, chrysin 6-*C*-glucoside-8-*C*-arabinoside, scutellarin, quercetin, baicalin, wogonin 7-*O*-glucuronide (wogonoside), oroxylin A-7-*O*-glucuronide (oroxyloside), rhamnocitrin, baicalein, and oroxylin A (Figure 4a). Moreover, pure baicalein was the strongest signal in the analysis (Figure 4b), and baicalin was the second strongest signal in the analysis (Figure 4c). The major component, retention time (RT), measured *m*/*z*, calculated *m*/*z*, molecular formula, intensity, and the relative abundance percentage are listed in Table S1. All the characterizations of the major chemical components in *S. baicalensis* were confirmed before being further subjected to biological evaluation. According to the experimental results, we investigated the efficacy of baicalein and baicalin in inhibiting pseudovirus infection.

### 2.5. Baicalein and Baicalin Inhibition of Omicron Spike Pseudovirus Infection

**Figure 5:**
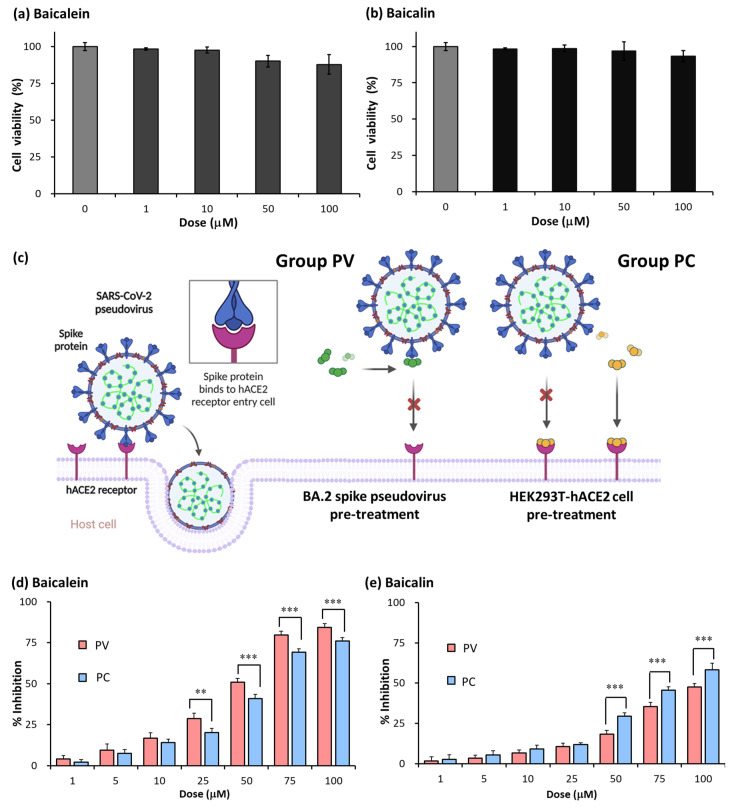
Assessment of inhibitory efficacy of baicalein and baicalin against SARS-CoV-2 BA.2 spike pseudovirus infection. The viability of HEK293T-hACE2 cells treated with baicalein was quantified via MTT assay (**a**). The viability of HEK293T-hACE2 cells treated with baicalin was quantified via MTT assay (**b**). Experimental scheme indicating the pseudovirus pretreatment (PV group) and the cell pretreatment (PC group) of baicalein and baicalin (**c**). The inhibitory efficacy of baicalein pretreated with SARS-CoV-2 BA.2 spike pseudovirus and HEK29T-hACE2 cells was quantified using a Synergy HT multimode microplate reader (**d**). The inhibitory efficacy of baicalin pretreated with SARS-CoV-2 BA.2 spike pseudovirus and HEK29T-hACE2 cells was quantified using a Synergy HT multimode microplate reader (**e**). Red color (PV group): baicalein and baicalin pretreated with SARS-CoV-2 spike pseudovirus. Blue color (PC group): baicalein and baicalin pretreated with HEK293T-hACE2 cells. All values are expressed as mean ± SD for each group (*n* = 5). The statistical comparison between groups was conducted using Student’s *t*-test; ** *p* < 0.01 and *** *p* < 0.001 vs. PV group.

Based on the results from the LC-MS analysis, baicalein and baicalein were the main active components in *S. baicalensis*. To ensure optimal experimental conditions, we initially measured the cell viability of baicalein and baicalein at final concentrations of 0, 1, 10, 50, and 100 μM. The results show that their similarities in terms of cell viability did not change after treatment with baicalein (Figure 5a) or baicalin ≤ 100 μM (Figure 5b). We further examined the inhibitory activities of baicalein and baicalin. The pseudovirus system was used to elucidate the affinity between the spike protein, hACE2, baicalein, and baicalin. To better understand their inhibitory activities, we examined baicalein and baicalin pretreated with SARS-CoV-2 spike pseudovirus (PV group) and HEK293T-hACE2 cells (PC group) for 4 h (Figure 5c). We used different concentrations (1, 5, 10, 25, 50, 75 μM) of baicalein and baicalin in the experiment to determine their inhibition efficacy. For baicalein, the inhibitory ratios in the PV group were 4.1, 9.4, 16.7, 28.8, 50.9, 79.8 and 84.3%, respectively. The inhibitory ratios in the PC group were 2.1, 7.5, 14.1, 20.3, 40.9, 69.3 and 76.1%, respectively (Figure 5d). For baicalin, the inhibitory ratios in the PV group were 1.7, 3.5, 6.7, 10.7, 18.3, 35.3, and 47.5%, respectively. The inhibitory ratios in the PC group were 2.6, 5.4, 9.0, 11.9, 29.5, 45.6 and 58.3%, respectively (Figure 5e). These results indicate the dose-dependent inhibitory effect of baicalein and baicalin on spike protein pseudovirus infection. Additionally, these results showed that the inhibitory efficacy of baicalein can be strengthened via pretreatment with pseudovirus. On the contrary, the inhibitory efficacy of baicalin can be strengthened via pretreatment with HEK293T-hACE2 cells.

### 2.6. Synergistic Effect of Baicalein and Baicalin on Pseudovirus Infection Inhibition

**Figure 6:**
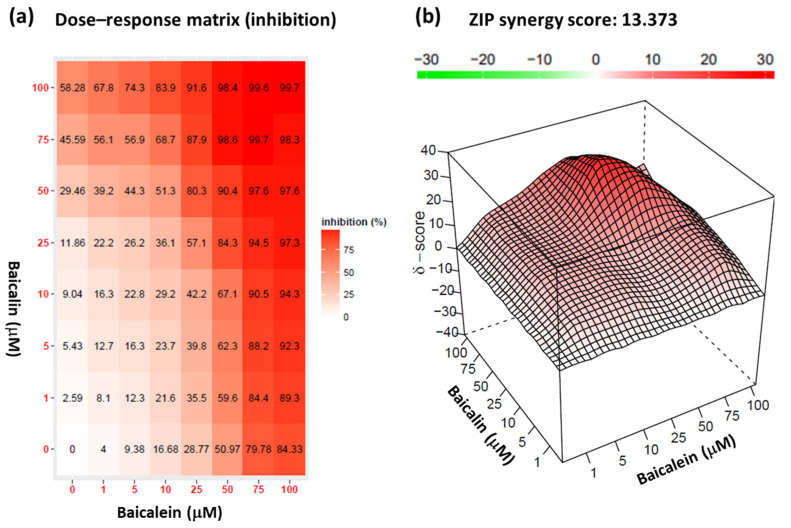
Synergism between baicalin and baicalein. Dose–response map for a synergistic drug combination (baicalein and baicalin) (**a**). ZIP synergy score map for the same drug combination (**b**). The interaction results are shown in both 2D and 3D. δ, excess % inhibition beyond the expectation predicted with the ZIP model.

To identify an effective strategy for SARS-CoV-2 prevention, targeting the interaction of mutated spike proteins with hACE2 receptors, we investigated the synergistic effect between baicalein and baicalin. We combined baicalein pretreated for 4 h with the pseudovirus and baicalin pretreated with HEK293T-hACE2 cells at final concentrations of 0, 1, 5, 10, 25, 50, 75, and 100 μM. We used a zero-interaction potency (ZIP) synergy score map to visualize the synergistic patterns between baicalein and baicalin. As shown in Figure 6a, the inhibitory value for the responses varied at different doses, suggesting that not all scores were equally important. High delta scores (>5) were also confirmed for various combinations of baicalein and baicalin. When 25 µM baicalein was combined with 75 µM baicalin, 87.9% virus inhibition was attained. However, the inhibition rate was only 84.3% for 100 µM baicalein. The interaction of baicalein and baicalin was synergistic over the dose–response matrix, leading to a maximal combination effect of close to 100% cell inhibition at higher concentrations of both drugs (Figure 6b). The average delta score for the confirmed combinations was 13.373, which was significantly higher than the average synergy score (=10) of the combinations. Taken together, the interaction results indicated that the combination of baicalein and baicalin could produce a stronger effect than individual compounds while maintaining acceptable doses and limiting the side effects.

## 3. Discussion

TCM is an important source of bioactive natural products that are widely used for the treatment of hepatitis, atherosclerosis, hypertension, hyperlipidemia, type 2 diabetes, and respiratory disorders [30,31,32]. *S. baicalensis* has been widely used in TCM for COVID-19 treatment. *S. baicalensis* is an effective screening object when aiming to inhibit SARS-CoV-2 infection [33,34,35,36]. However, the major components of *S. baicalensis* and their mechanisms remain to be explored. 

The challenge in studying SARS-CoV-2′s pathogenicity is the limited availability of biosafety level 3 (BSL-3) facilities. The inhibitory effects of the selected TCM on viral entry, a pivotal step in virus infection, were determined using a pseudovirus system (Figure 1). This method does not require live biological materials or biosafety containment. In the present study, an Omicron variant spike-protein-expressing pseudovirus was established to evaluate the binding efficacy of variant spike proteins to hACE2 receptors (Figure 2). *S. baicalensis* was the most effective TCM herb in NRICM101 in terms of inhibiting the Omicron spike protein pseudovirus (Figure 3). Baicalein and baicalin are the main components of *S. baicalensis* (Figure 4), which both inhibit SARS-CoV-2′s infection of cells via the hACE2 receptor. Baicalein preferentially blocks the viral spike protein, whereas baicalin preferentially blocks hACE2 (Figure 5). In addition, the interaction results indicated the efficacy of the combination of baicalein and baicalin in targeting the interaction between the spike protein and ACE2 (Figure 6). 

TCM has been widely applied to treat COVID-19 and was found to alleviate the symptoms of the disease, delay the progression of mild to severe infection, and increase cure rates [35,36]. NRICM101 has demonstrated both antiviral and anti-inflammatory effects against COVID-19 since 2020 [16,23], showing promise as a multitarget agent for the prevention and treatment of COVID-19 [16]. However, given the high mutation rate of the virus and the increased risk of immune viral escape, emerging SARS-CoV-2 variants have exhibited greater antiviral resistance, allowing the virus to escape the immune system [37,38]. Thus, this study aimed to explore the more effective components in the TCM used to treat SARS-CoV-2 variants by targeting the interaction between mutated spike proteins and hACE2. 

Some of the TCM herbs in NRICM101 have anti-inflammatory effects, such as *H. cordata* and *I. indigotica* [39,40]; some have antioxidant effects, such as mulberry leaf [41]. Specifically, *S. baicalensis* exerts antiviral effects by inhibiting the activation of 3C-like protease (3CLpro) and the replication of SARS-CoV-2 [22,42]. Most studies have indicated the antiviral ability of *S. baicalensis* on the spike protein/ACE2 interaction through molecular docking approaches [42,43]. There is a lack of in vitro and in vivo evidence directly indicating that the main competent of *S. baicalensis* has inhibitory effects on the interaction between the spike protein and hACE2. Thus, the purpose of this study is to enhance the current understanding of the inhibitory roles of the main components of *S. baicalensis* in the spike protein/hACE2 interaction by conducting in vitro experiments. In the present study, the inhibition of the spike protein/hACE2 interaction by *S. baicalensis* provided direct evidence demonstrating the inhibitory efficacy of this herb (Figure 3). In addition, we performed an LC-MS fingerprint analysis to reveal the active ingredients of *S. baicalensis*, which include baicalin, baicalein, wogonin, wogonoside, and oroxylin A. 

According to the LC-MS data, baicalein and baicalin are the major and secondary components of *S. baicalensis*, respectively (Figure 4). Baicalein and baicalin possibly play crucial roles in the prevention of SARS-CoV-2 invasion. Previous studies have indicated that baicalein and baicalin have various pharmacological effects, including anti-inflammatory, antiviral, antibacterial, hepatoprotective, and choleretic activities [44,45]. These compounds were shown to be potential inhibitors in the RNA replication process of the virus [46,47] and of the crucial 3CLpro in SARS-CoV-2 [48,49]. However, no experiments have been conducted targeting the spike protein/hACE2 interaction for the prevention of SARS-CoV-2 invasion. Previous computational results showed that baicalein and baicalin potentially have a binding affinity to the spike protein and hACE2 enzyme, respectively [50,51]. The present study provides in vitro results to prove the inhibitory efficacy of baicalein and baicalin on the spike protein/hACE2 interaction.

Baicalein forms a stable hydrogen bond with the spike protein Gln498, a key amino acid residue, in the binding of the spike protein to the hACE2 receptor [24]. The results of a molecular docking analysis showed that baicalin strongly binds with hACE2, with a binding energy of −8.46 kcal/mol, via binding to the residues Asn149, Arg273, and His505 of hACE2 [50]. In this study, the in vitro pseudovirus results support the published bioinformatics and molecular interaction results. Baicalein had a stronger inhibitory efficacy when pretreated with the SARS-CoV-2 spike pseudovirus. Baicalin had a higher inhibitory efficacy when pretreated with HEK293T-hACE2 cells (Figure 5). Based on the above findings, we consider baicalein to be a potential spike blocker and baicalin as an ACE2 blocker mediating the interaction between the viral spike protein and hACE2. According to the results of a pharmacological effect assay and dynamics simulations, Song et al. [51] reported that baicalein can simultaneously interfere with multiple targets, as well as with the interaction between the spike protein and the hACE2 receptor. Baicalein may be a potential candidate for the treatment of SARS-CoV-2 and its variants, especially the Omicron variant. For the hACE2/S-RBD interaction in the original strain, the virus, baicalein, interacts with the active site through three hydrogen bonds with Asn33, Tyr505, and Arg393, showing van der Waals interactions with the Asp30 and Glu37 of the spike protein. For the hACE2/S-RBD of the Delta variant, Song et al. [51] found that baicalein interacts with Asp30, Lys26, Leu29, Asn33, Glu96, Pro389, Gln388, Ala387, Thr92, Val93, and Asn90 residues. Shakhsi-Niaei et al. [52] reported that baicalin is a candidate that is able to occupy hACE2 and inhibit viral RBD binding, i.e., baicalin is a strong inhibitor of the SARS-CoV-2 binding site of ACE2. The results of this molecular simulation study showed that baicalin interacts with the active site through three hydrogen bonds with A396, E208, and D206. Many scientists are currently studying the possibility of developing molecules, for example, those based on baicalein and baicalin from *S. baicalensis*, as effective antiviral drugs [24,33,50,51,52].

To identify an effective strategy for SARS-CoV-2 prevention, we investigated the synergistic effects between baicalein and baicalin. An efficient experimental–computational approach was used to identify the most potent synergistic and antagonistic combinations of these two compounds [31,53]. In this study, we systematically conducted combination experiments to assess drug interactions, using a ZIP model to capture shifts in interaction potency [54]. To conduct a more systematic analysis of the whole dose–response matrix, we implemented a surface plot approach based on delta scoring to visualize the range of drug interactions over all tested dose pairs. The interaction results indicate that the combination of baicalein and baicalin produced stronger effects than the individual compounds while maintaining acceptable doses and limited side effects. Further studies are required to analyze these compounds and their structurally related derivatives. The increased specificity of combinations of baicalein and baicalin has implications for the development of anti-SARS-CoV-2 drug candidates for future preclinical studies. Although the current study provides the first in vitro evidence that baicalein and baicalin have inhibitory effects on the interaction between the viral spike protein and the hACE2 receptor, other extracts from *S. baicalensis* may also play a potential role in the inhibition of viral infection. In the future, the detailed pathogeneses and mechanisms of other extracts from *S. baicalensis* should be further investigated as potential drugs for COVID-19 treatment.

## 4. Materials and Methods

### 4.1. Chemicals and Reagents

Anti-ACE2 polyclonal antibody (N1N2) (Cat#GTX101395), anti-SARS-CoV/SARS-CoV-2 (COVID-19) spike monoclonal antibody (1A9) (Cat#GTX632604), goat anti-rabbit IgG antibody (Cat#GTX213110-01) and goat anti-mouse IgG antibody (Cat#GTX213111-01) were purchased from Genetex (Irvine, CA, USA). Anti-β-actin rabbit monoclonal antibody (Cat#GTX109639) was purchased from Cell Signaling Technology (Danvers, MA, USA). Baicalein (5,6,7-trihydroxyflavone) (Cat#HY-N0196) and baicalin (5,6,7-trihydroxyflavone-7-*O*-β-d-glucuronide) (Cat#HY-N0197) were purchased from MedChemExpress (Princeton, NJ, USA).

### 4.2. Cell Culture and Cell Viability Assay

The human embryonic kidney 293T cell line (HEK293T) was obtained from American Type Culture Collection (ATCC; Manassas, VA, USA). HEK293T cells stably expressing hACE2, named the HEK293T-hACE2 cell line, were obtained from the Food Industry Research and Development Institute (Hsinchu, Taiwan). HEK293T and HEK293T-hACE2 cells were cultured in Dulbecco’s modified Eagle’s medium (DMEM; Thermo Fisher Scientific, Waltham, MA, USA) with 10% fetal bovine serum (FBS) and maintained in a humidified atmosphere of 5% CO_2_ at 37 °C.

For the cell viability assay, the HEK293T-hACE2 cells were seeded in 96-well plates and incubated for 24 h before starting the experiment. The cells were cultured with treatment for 24 h. After the treatment, 10 μL of 3-(4,5-Dimethyl-2-thiazolyl)-2,5-diphenyl-2*H*-tetrazolium bromide (MTT) solution was added to each well for 4 h. Then, all fluid in the wells was aspirated and 100 μL of DMSO was added to each well, which was then shaken for 10 min. Afterward, GPF fluorescence was detected using a Synergy HT multimode microplate reader (BioTek Instruments, Winooski, VT, USA), and absorbance was detected at 540 nm.

### 4.3. Pseudovirus System

**Figure 7:**
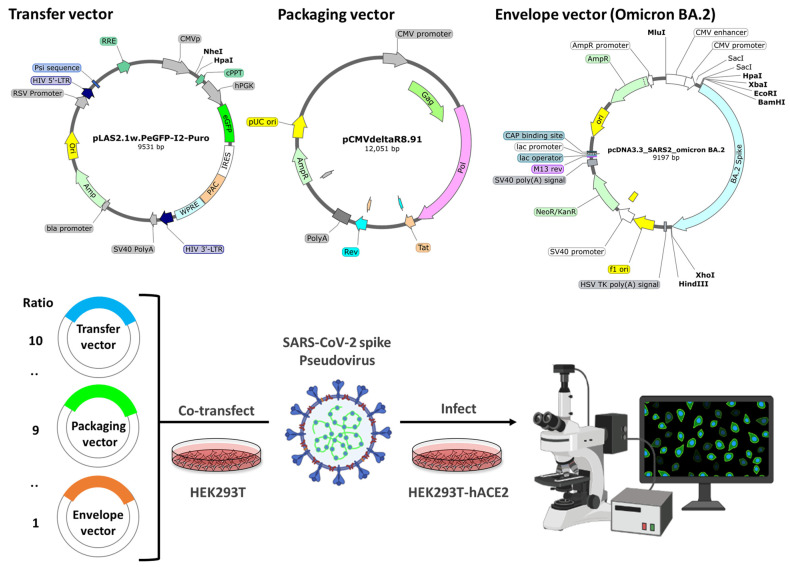
The SARS-CoV-2 pseudovirus system construction. The transfer vector is pLAS2.1w.FLuc-I2-Puro, which expresses the eGFP gene. The packaging vector is pCMVΔR8.91, which expresses HIV-1 Gag and polymerase gene. The envelope vector is pcDNA3.3_SARS2_omicron BA.2, which expresses Omicron BA.2. The plasmid DNA were co-transfected into HEK293T cells to produce the pseudovirus. The GFP of the infected cells was observed via microscopy at 72 h after infection.

As shown in Figure 7, the lentivirus packaging system was used to produce the SARS-CoV-2 spike pseudovirus. Three vectors were chosen for construction: pCMVΔR8.91, pLAS2.1w.PeGFP-I2-Puro (RNAiCore, Taipei, Taiwan), pcDNA3.1-SARS2-Spike, and pcDNA3.3_SARS2_Omicron BA.2 (Addgene, Watertown, MA, USA). HEK293T was seeded onto a 24-well plate and cultured for 24 h. Then, 15 μg of plasmid DNA (ratio of transfer vector: packaging vector/envelope vector = 10:9:1) and Lipofectamine 2000 Transfection Reagent (Thermo Fisher Scientific) were co-transfected into HEK293T cells. The pseudovirus was harvested 72 h after transfection for the experiments. The HEK293T-hACE2 cells were infected with the pseudovirus for 24 h at 37 °C. After the incubation, the virions were removed, and the infected cells were cultured in fresh medium for another 72 h. The GFP of the cells was observed via microscopy at 72 h after infection. Additionally, the cells were collected 72 h after infection to analyze the GFP intensity using a Synergy HT multimode microplate reader (BioTek Instruments).

### 4.4. Pseudovirus Infection Inhibition Assay

HEK293T-hACE2 cells were seeded into 24-well plates (5 × 10^4^ cells/well) 24 h before the experiment. The blocker molecules or TCM extracts were co-incubated with the pseudovirus for 24 h at 37 °C. The next day, the pseudovirus was removed, and cells were cultured in the fresh medium for another 72 h. To evaluate the inhibition efficiency, the GFP intensity was measured 72 h after infection, and the treatment groups were compared with the control group.

### 4.5. Protein Extraction

The cell samples derived from HEK293T and HEK293T-hACE2 cells were collected and then homogenized with PRO-PREPTM protein extraction solution (INTERCHIM, San Diego, CA, USA). The cell lysate was centrifuged at 13,000× *g* for 10 min, and the supernatants were collected and preserved. We quantified the total protein in the cell lysate using a Bradford dye binding assay (Bio-Rad Laboratories, Hercules, CA, USA) with bovine serum albumin as the standard. The cell protein lysate was aliquoted and stored at -25 °C until further use.

### 4.6. Western Blot

Samples containing 10 μg of protein with 4X protein dye were loaded onto 10% SDS-PAGE gels. After electrophoretic separation, polyvinylidene fluoride membranes (PVDF; Bio-Rad Laboratories) were soaked in methanol for 1 min. Then, the protein sample was transferred to a PVDF membrane via wet electroblotting. To block nonspecific binding, the PVDF membranes were treated with a blocking solution consisting of 5% nonfat milk in Tris-buffered saline. The primary antibodies used for the ACE2 receptor, spike protein, and β-actin were diluted to 1:1000. The secondary antibodies were diluted to 1:4000. The substrate containing secondary antibodies was conjugated horseradish peroxidase (HRP) and enhanced chemiluminescent (ECL) was used to detect HRP (GeneTex). The target location of the bands was detected based on protein weight using iBright CL750 Imaging Systems (Thermo Fisher Scientific). The band’s intensity was quantified using the original ImageJ (NIH, Bethesda, MD, USA). The expression levels of hACE2 and spike proteins were calculated and compared with that of β-actin for every sample.

### 4.7. Extraction of Each TCM

Dried *Scutellaria* root, heartleaf, and *Indigowoad* root (60 g) were purchased from a Chinese herbal store in Taipei, Taiwan, and were authenticated by Dr. Pei-Ching Wu at the Department of Chinese Medicine, China Medical University Hospital, Taichung, Taiwan. The herbs were chopped and boiled in 600 mL of water for 30 min and simmered until the decoction reduced to 100 mL. The supernatant was collected and filtered through a 0.22 μm PVDF filter. The filtered solution was aliquoted and stored at −80 °C for 1 day. Then, the filtered solution was dried in a freeze-dryer for 4–5 days to yield a crude extract. The lyophilized powder of the extracts (20 mg) was dissolved in 1 mL of water as a stock solution (20 mg/mL). All the tested solutions were filtered through a 0.22 μm PVDF filter before use. 

### 4.8. LC-MS Analyses

LC-MS analyses were conducted by the Center for Advanced Instrumentation and the Department of Applied Chemistry at National Yang Ming Chiao Tung University, Hsinchu, Taiwan. Ultra-performance liquid chromatography (UPLC) of the sample solutions (pH 7.5) was performed via direct infusion (2 μL). High-resolution electrospray mass spectrometry (HRESI-MS) was conducted with an Impact HD Q-TOF mass spectrometer (Bruker, Karlsruhe, Germany) that was equipped with an electrospray ionization (ESI) source in positive-ion mode. The detailed ESI(+) parameters were as follows: the ion spray voltage was 4.5 kV; the capillary temperature was 200 °C; and the sheath gas flow rate was 6 L/min. The mass spectra were collected over the mass range of *m*/*z* 50–1500 at a resolving power of 40,000. The final data were analyzed using Compass DataAnalysis 4.1 (Bruker). A 20 mg/mL solution of the compound was analyzed.

### 4.9. Synergy Scoring and Detection

The synergic effect of baicalein and baicalin was predicted using SynergyFinder 3.0, which is a web application for analyzing dose–response matrix data of drug combinations [55]. Cells were treated with baicalein at a final concentration of 1, 5, 10, 25, 50, 75, and 100 μM and with baicalin at 1, 5, 10, 25, 50, 75, and 100 μM for 4 h. The cells were collected 72 h after infection to analyze the GFP intensity using a Synergy HT multimode microplate reader. The Bliss independence model was used as the primary method to score drug combination synergy in the datasets; although the results are based on the Loewe additivity, the results of the highest single agent (HSA) and ZIP model are additionally shown to provide complementary information from various models [56]. A synergy score >10 is considered synergistic, a score of between −10 and +10 is considered additive, and a synergy score < −10 is considered antagonistic [57].

### 4.10. Statistical Analysis

All values are expressed as the mean ± standard deviation (SD; *n* = 3 for each treatment). The data were compared using Student’s *t*-test to evaluate differences between groups. *p*-value ≤ 0.05, *p*-value ≤ 0.01, and *p*-value ≤ 0.001 were considered statistically significant.

## 5. Conclusions

This study systematically evaluated the antiviral activity of TCM using a SARS-CoV-2 spike pseudovirus system. We identified two natural components, baicalein and baicalin, as novel antiviral therapeutic candidates for blocking the interaction between the viral spike protein and the host hACE2 receptor, thereby inhibiting SARS-CoV-2 infection. Our results warrant further evaluation and characterization of these molecules to work toward the development of a drug for COVID-19 treatment. Baicalein bound to the viral spike protein with a stronger affinity, while baicalin bound to the hACE2 receptor with a stronger affinity. In addition, the drug synergy results indicated that the combination of baicalein and baicalin produced a stronger effect than either compound alone while maintaining acceptable doses and limited side effects. Further studies are needed to analyze these compounds and their structurally related derivatives, alone and in combination with effective nucleoside analogs, for the development of novel anti-SARS-CoV-2 drug candidates for preclinical studies. The mechanisms and dynamic reactions of baicalein and baicalin in combating viral infections remain to be explored in more detail.

## Figures and Tables

**Table 1:** Groups in pseudovirus system with putative viral spike protein blocker and putative hACE2 blocker treatments.

Groups	Treatment
SPV	Spike blocker (Cysteamine) pre-treated with SARS-CoV-2 spike pseudovirus
SPC	Spike blocker pre-treated with HEK293T-hACE2 cells
APV	hACE2 blocker (Dalbavancin) pre-treated with SARS-CoV-2 spike pseudovirus
APC	hACE2 blocker pre-treated with HEK293T-hACE2 cells

HEK293T-hACE2 cells were infected with SARS-CoV-2 spike pseudovirus. A putative viral spike protein blocker (cysteamine) and a putative hACE2 blocker (dalbavancin) pre-treated with SARS-CoV-2 spike pseudovirus and HEK293T-hACE2 cells, respectively.

## Data Availability

The data presented in this study are available upon request from the corresponding author.

## Supplementary Materials

The supporting information can be downloaded at: https://www.mdpi.com/article/10.3390/ijms25052935/s1.
