# Supplementary material for: Inhibitory Efficacy of Main Components of Scutellaria baicalensis on the Interaction between Spike Protein of SARS-CoV-2 and Human Angiotensin-Converting Enzyme II
Source: Int J Mol Sci. 2024 Mar 2;25(5):2935. doi: 10.3390/ijms25052935 (PMC10932139; doi:10.3390/ijms25052935)
Supplement: Supplementary file 1 [file ijms-25-02935-s001.zip › ijms-2869344-supplementary.pdf]

Table S1. LC-MS analysis of constituents in *S. baicalensis.*

Peak No.	Major Component	Retention Time (min)	Measured *m/z*	Calculated *m/z*	Molecular Formula	Intensity	Percentage (%)
1	Chrysin 6-*C*-arabinoside-8-*C*-glucoside	2.5	547.1323	547.1457	$C_{26}H_{27}O_{13}$	4,919,767	13.1
2	Chrysin 6-*C*-glucoside-8-*C*-arabinoside	3.2	547.1318	547.1457	$C_{26}H_{27}O_{13}$	3,280,141	8.3
3	Scutellarin	3.5	461.1123	461.0803	$C_{21}H_{18}O_{12}$	315,107	2.1
4	Quercetin	5.4	301.0262	301.0432	$C_{15}H_{10}O_{7}$	370,341	0.7
5	Baicalin	7.8	445.0662	445.0776	$C_{21}H_{17}O_{11}$	4,759,367	16.5
6	Wogonoside	8.0	459.0819	459.0933	$C_{22}H_{19}O_{11}$	3,733,587	6.9
7	Oroxyloside	8.2	459.0818	459.1.011	$C_{22}H_{20}O_{11}$	4,646,815	10.1
8	Rhamnocitrin	8.9	299.0475	299.0612	$C_{16}H_{12}O_{6}$	724,107	1.4
9	Baicalein	9.3	269.0376	269.0455	$C_{15}H_{9}O_{5}$	14,651,640	34.2
10	Oroxylin A	10.3	283.0528	283.0612	$C_{16}H_{11}O_{5}$	1,607,887	8.1

Note. "*m*" represents the mass and "*z*" represents the charge of the ion. *m/z* represents the mass divided by the charge number, known as the mass-to-charge ratio, and is used as the horizontal axis unit in the mass spectrum.
